# Birthweight, childhood growth and left ventricular structure at age 60–64 years in a British birth cohort study

**DOI:** 10.1093/ije/dyw150

**Published:** 2016-07-13

**Authors:** Rebecca Hardy, Arjun K Ghosh, John Deanfield, Diana Kuh, Alun D Hughes

**Affiliations:** 1 MRC Unit for Lifelong Health and Ageing at UCL, London, UK; 2 Barts Heart Centre, London, UK; 3 International Centre for Circulatory Health, Imperial College London, UK and; 4 Institute of Cardiovascular Science, University College London, UK

**Keywords:** Birthweight, growth, overweight, left ventricular structure, birth cohort, life course

## Abstract

**Background:** High left ventricular mass (LVM) is an independent predictor of cardiovascular disease and mortality, but information relating LVM in older age to growth in early life is limited. We assessed the relationship of birthweight, height and body mass index (BMI) and overweight across childhood and adolescence with later life left ventricular (LV) structure.

**Methods:** We used data from the MRC National Survey of Health and Development (NSHD) on men and women born in 1946 in Britain and followed up ever since. We use regression models to relate prospective measures of birthweight and height and BMI from ages 2–20 years to LV structure at 60–64 years.

**Results:** Positive associations of birthweight with LVM and LV end diastolic volume (LVEDV) at 60–64 years were largely explained by adult height. Higher BMI, greater changes in BMI and greater accumulation of overweight across childhood and adolescence were associated with higher LVM and LVEDV and odds of concentric hypertrophy. Those who were overweight at two ages in early life had a mean LVM 11.5 g (95% confidence interval: -2.19,24.87) greater, and a mean LVEDV 10.0 ml (3.7,16.2) greater, than those who were not overweight. Associations were at least partially mediated through adult body mass index. Body size was less consistently associated with relative wall thickness (RWT), with the strongest association being observed with pubertal BMI change [0.007 (0.001,0.013) per standard deviation change in BMI 7–15 years]. The relationships between taller childhood height and LVM and LVEDV were explained by adult height.

**Conclusions:** Given the increasing levels of overweight in contemporary cohorts of children, these findings further emphasize the need for effective interventions to prevent childhood overweight.

Key Messages

- High left ventricular mass is independently associated with cardiovascular disease, and changes in left ventricular structure have been observed in overweight children.
- Associations between increasing birthweight and higher left ventricular mass and end diastolic volume are explained by adult height, and thus largely reflect normal somatic growth.
- High BMI and overweight in childhood and adolescence and accumulation of overweight across the life course are associated with greater left ventricular mass and left ventricular end diastolic volume, with associations being at least partially mediated through adult body mass index.
- Given the increasing levels of overweight in contemporary cohorts of children, the need for effective interventions to reduce childhood overweight is highlighted, in order to prevent altered left ventricular structure.

## Introduction

Left ventricular hypertrophy (LVH) and high left ventricular mass (LVM) are independent predictors of adult cardiovascular morbidity and mortality.^1^ LV structure changes with age and is thus an important intermediate marker of disease risk. Studies have documented tracking of body size and LVM across childhood,^2–4^ and adiposity in childhood is one of the two major factors [blood pressure (BP) being the other] that lead to excessive cardiac growth beyond that expected to accompany normal somatic growth in children.^5^ In the Generation R study, overweight and obese children have been found to show cardiac adaptations by the age of 2 years.^6^ The same study also found higher birthweight, a marker of fetal growth, to be associated with higher LVM at age 2 years.^7^ Similar associations have been observed in studies with LVM measured at age 9 years^8^ and 15 years,^9^ although associations were attenuated after adjustment for current height and weight.

The few existing studies investigating the longer-term associations of prenatal and postnatal growth have found no association between birthweight and LVM in adulthood.^10–12^ In contrast, another study has reported that being born preterm was associated with higher LVM indexed to body surface area in young adulthood.^13^ There is also evidence of an association between higher weight in early postnatal life and lower LVM in adulthood.^11^^,^^12^ However, these existing studies included samples of less than 500 and may therefore have lacked the power to detect relatively small associations, and did not assess adiposity development past infancy.

In the Bogalusa Heart Study, adiposity beginning in childhood was related to higher LVM index (LVMI) and LVH in early adulthood.^14–16^ A few other studies suggest that high body mass index (BMI) in adolescence or early adulthood is associated with higher LVM up to 20 years later.^17^^,^^18^ However, these existing studies have not considered measures of body size from different stages of early life in the same individuals, to investigate possible sensitive periods of development when the accumulation of adiposity may have particularly detrimental effects. Short adult height (and particularly short leg length) represents the impact of environmental factors in early childhood,^19^ and has consistently been associated with adverse cardiovascular outcomes and mortality.^20^

To our knowledge there are no studies that assess the associations between childhood and adolescent BMI and height change, and echocardiographic measures past mid life. Most research has focussed on BMI and adiposity in early life, and information relating height growth to cardiac structure in adulthood is scarce.

Using data from the MRC National Survey of Health and Development (NSHD), a birth cohort study of men and women born in Britain in 1946, we aimed first to assess the relationship of birthweight and subsequent childhood and adolescent change in height and BMI with LV structure at age 60–64 years, assessing whether height and BMI in earlier life were associated with LV structure over and above the association with current body size. We assessed whether particular periods of growth were more strongly related with the outcomes and whether greater accumulation of overweight across childhood and adolescence was associated with LV structure. Finally, we assessed whether any observed associations were mediated by adult cardiovascular (CV) risk factors.

## Methods

The Medical Research Council (MRC) National Survey of Health and Development (NSHD) is based on a nationally representative sample of 5362 births out of all the single births to married mothers that occurred in 1 week in March 1946 in England, Scotland and Wales.^21^ The cohort has been followed up 23 times, with the most recent data collection in 2006–10 when participants were 60–64 years old.^22^ Study members still alive and with a known current address in England, Scotland or Wales were invited for an assessment at one of six clinical research facilities (CRF) or to be visited by a research nurse at home. Invitations were not sent to those who had died (*N* = 778), who were living abroad (*N* = 570), had previously withdrawn from the study (*N* = 594) or had been lost to follow-up (*N* = 564). Of the 2856 invited participants, 2229 (78%) were assessed: 1690 (59%) attended a CRF and the remaining 539 were visited at home. The participating sample remains broadly representative of native-born British men and women of the same age.^23^

Ethical approval was obtained from the Greater Manchester Local Research Ethics Committee and the Scotland Research Ethics Committee. Written informed consent was obtained from the study members for each component of each data collection.

### Echocardiographic measurement

Of the 1690 participants who attended a clinic, 1653 underwent echocardiography using GE Vivid I machines and 1617 had analysable images (96%). Echocardiographic images were obtained from parasternal long axis and short axis, apical 5-chamber, 4-chamber, 3-chamber, 2-chamber and aortic views along with conventional and tissue Doppler in the 4-chamber view. Image analysis was carried out by a cardiologist (A.K.G.) and two experienced cardiac physiologists masked to participant identity, using GE EchoPac software. Wall and chamber measurements were made according to American Society of Echocardiography (ASE)/European Association of Echocardiography (EAE) guidelines. LVM, LV end diastolic volume (LVEDV) and relative wall thickness (RWT) were calculated using the ASE-recommended formulae.^24^ A categorical variable representing LV remodelling was derived and the normal group [normal LVMI indexed for body surface area (BSA) and normal RWT] was compared with eccentric LVH (increased LVMI and normal RWT), concentric LVH (increased LVMI and increased RWT) and concentric remodelling (normal LVMI and increased RWT).^24^

Echocardiographers and readers underwent standardized training, periodic review and refresher sessions; phantom scans were performed annually on all machines and a continuous quality control audit of scans with feedback was carried out. A reproducibility study found excellent inter- and intra-reader reproducibility (intra-class correlation coefficients were > 0.9 for most measurements).

### Anthropometry and covariates

Birthweights, recorded to the nearest quarter of a pound, were extracted from medical records. Weight and height measured at ages 2, 4, 6, 7, 11, 15 and 60–64 years and self-reported at 20 years were used to calculate BMI. Overweight or obese and underweight at each age was defined according to age-specific cut-offs.^25^

Childhood socioeconomic position (SEP) was measured by father's occupational social class and categorized according to the Registrar General's classification, and adult SEP by own occupation at age 53 years. Sitting heart rate (HR) and brachial blood pressure (BP) were measured using an Omron HEM-705 with an appropriately sized cuff after a 5-min rest. The second measurement was used in analyses, or the first measure where the second was missing. Type 2 diabetes status was based on self-reports of doctor diagnosis.

### Statistical methods

Regression models were used to separately relate birthweight and each pair of childhood and adolescent BMI and height to LVM, RWT and LVEDV. Tests for sex x body size interactions were also carried out; there was no evidence of any sex differences in any association, so all are presented from sex combined models with an adjustment for sex. Childhood and adult SEP were then added to the models and birthweight for the BMI and height models. Additional adjustments for current BMI and height were added; non-linear terms and sex interactions were used where appropriate after investigation in preliminary models including only adult height, BMI and sex.

In order to assess the impact of differential growth over the period of childhood and adolescence, conditional change in BMI and height between the selected ages of 2–4, 4–7, 7–15 and 15–20 years was calculated. The conditional change variables were derived by regressing BMI (or height) at time (t) on BMI (or height) at t-1 for each sex and extracting standardized residuals. Analyses were adjusted for the confounders and then additionally for CV risk factors. In order to assess accumulation of overweight, we selected ages 4 (early childhood), 7 (later childhood) and 15 (adolescence) to represent different stages of early life. We defined the number of times (0–3) that an individual had been recorded as overweight or obese, and related this variable to the outcomes. Adjustments were carried out for confounders, and then current height and overweight were added in order to assess whether overweight in childhood and adolescence is associated with LV outcomes even among normal weight adults. Subsequently, CV risk factors were also adjusted for in a model without current body size.

Sensitivity analyses were carried out, repeating the analyses with LVMI and LVEDVI (indexed first to BSA and second to height^2^^,^^7^) as the outcomes. In these models, adjustment was not made for body size as body size is already accounted for by the indexation.

Finally, analyses were repeated for LV remodelling as the outcome using multinomial logistic regression. There was insufficient statistical power to adequately investigate accumulation of overweight and LV remodelling.

## Results

A total of 782 men and 835 women had at least one outcome measure and at least one early life body size measure; adult BMI and height and descriptive statistics are provided in Table 1. In this maximum analytical sample, mean LV measures were all higher in men than women. The percentage of men and women with concentric and eccentric hypertrophy was similar, but a slightly higher percentage of men had concentric remodelling. In the total sample contacted at age 60–64, those included in these analyses were taller throughout life, had lower BMI at age 60–64 and were more likely to be from a non-manual social class in childhood and adulthood compared with those not included.

**Table 1.** Characteristics of the sample from the MRC National Survey of Health and Development with at least one echocardiographic outcome, adult body size and at least one early life body size measure

	Men			Women		
	*N* (%)	Mean	SD	*N* (%)	Mean	SD
Left ventricular mass (g)	700	209.2	60.3	775	156.0	45.7
Left ventricular end diastolic volume (ml)	762	112.4	29.8	804	84.6	20.2
Relative wall thickness	700	0.42	0.09	775	0.41	0.09
Left ventricular remodelling	700			775		
Normal	286 (40.9%)			343 (44.3%)		
Eccentric hypertrophy	99 (14.1%)			113 (14.6%)		
Concentric hypertrophy	113 (16.1%)			126 (16.3%)		
Concentric remodelling	202 (28.9%)			193 (24.9%)		
BMI at age 60–64y (kg/m^2^)	782	27.7	4.0	835	27.5	5.2
Height 60–64 (cm)	782	175.3	6.5	835	162.1	5.8
Birthweight (kg)	782	3.45	0.51	835	3.35	0.47
BMI 2y (kg/m^2^)	619	18.0	2.5	641	17.5	2.3
BMI 4y (kg/m^2^)	685	16.4	1.7	717	16.1	1.6
BMI 6y (kg/m^2^)	632	15.9	1.3	675	15.7	1.4
BMI 7y (kg/m^2^)	655	15.9	1.3	700	15.7	1.5
BMI 11y (kg/m^2^)	652	17.2	2.1	700	17.4	2.4
BMI 15y (kg/m^2^)	608	19.5	2.3	646	20.6	2.7
BMI 20y (kg/m^2^)	628	22.4	2.3	706	21.7	2.8
Height 2y (cm)	639	86.1	5.1	668	85.1	4.3
Height 4y (cm)	694	103.7	4.9	734	103.5	4.9
Height 6y (cm)	661	114.9	5.0	697	114.4	5.1
Height 7y (cm)	675	120.7	5.3	727	120.4	5.2
Height 11y (cm)	657	141.2	6.6	709	142.0	6.8
Height 15y (cm)	618	162.7	8.9	655	159.4	6.0
Height 20y (cm)	642	177.4	6.5	714	163.1	6.0
SBP 60–64y (mmHg)	780	139	18	833	133	18
DBP 60–64y (mmHg)	780	79	10	833	76	9
Resting heart rate 60–64y (beats/min)	780	67	11	833	70	11
Self-reported type 2 diabetes 60–64y	704			772		
No	667 (94.7%)			738 (95.6%)		
Yes	37 (5.3%)			34 (4.4%)		

y, years.

### Birthweight

Birthweight exhibited a non-linear relationship with LVM such that LVM increased with increasing birthweight, with a levelling off or even slight decrease at higher birthweights (Table 2). The association became more non-linear when adjusting for current BMI and height (Table 2). There was no evidence of an association between birthweight and LVMI indexed to either BSA or height^2^^,^^7^ (Supplementary Data, available as Supplementary Data at *IJE* online). LVEDV increased with increasing birthweight with the relationship being considerably attenuated after adjustment for current BMI and height (Table 3). Adjusting for BMI and height separately indicated that attenuation was a result of adjustment for height rather than BMI, a finding which is consistent with the null association observed with LVEDVI (Supplementary Data, available as Supplementary Data at *IJE* online). There was little evidence of a relationship between birthweight and RWT (Table 4).

**Table 2.** Mean difference (and 95% confidence interval) in left ventricular mass (g) by birthweight and each pair of BMI and height in childhood and adolescence: models adjusted for: sex only (model 1); sex and confounders (model 2); sex, confounders, and BMI and height at 60–64 years (model 3)

	Adjusted for sex (model 1)			Model 1 + additionally adjusted for childhood and adult social class and (except#) birthweight (model 2)			Model 2+ additionally adjusted for BMI, BMI x sex and height at 60–64 years (model 3)		
	Regression coefficient	(95% CI)	*P*-value	Regression coefficient	(95% CI)	*P*-value	Regression coefficient	(95% CI)	*P*-value
Birthweight# (per kg) (*n* = 1392)	6.4	(0.6,12.1)	0.03*	6.4	(0.7,12.1)	0.03*	1.7	(−3.7,7.0)	0.5*
Categories									
<2.5 kg	−9.1	(−26.8,8.7)	0.1	−11.0	(−28.7,6.8)	0.08	−8.3	(−24.3,7.8)	0.5
2.5–3.0 kg	−5.2	(−13.3,3.0)		−5.0	(−3.1,3.1)		−3.3	(−10.7,4.0)	
3.0–3.5 kg	Ref.			Ref.			Ref.		
3.5–4.0 kg	6.0	(−0.7,12.7)		6.1	(−0.6,12.8)		2.2	(−3.9,8.2)	
4.0–4.5 kg	2.6	(−8.7,13.9)		2.0	(−9.2,13.3)		−4.2	(−14.5,6.1)	
> = 4.5 kg	−2.0	(−21.7,17.6)		−2.1	(−21.6,17.5)		−3.5	(−21.2,14.2)	
BMI (per kg/m^2^)									
2 years (*n* = 1106)	2.1	(0.5,3.7)	0.01	2.2	(0.5,3.9)	0.01	0.5	(−1.1,2.1)	0.5
4 years (*n* = 1256)	3.6	(1.9,5.4)	<0.001*	3.4	(1.6,5.2)	<0.001*	1.5	(−0.1,1.3)	0.08*
Categories									
Lowest fifth	Ref.		<0.001	Ref.		<0.001	Ref.		0.09
2	6.6	(−2.3,15.5)		6.0	(−2.9,14.9)		0.0	(−8.1,8.0)	
3	8.9	(0.2,17.7)		8.4	(−0.4,17.2)		3.0	(−5.0,11.0)	
4	20.4	(11.2,29.7)		19.5	(10.3,28.8)		10.4	(1.9,18.8)	
Highest fifth	16.2	(7.2,25.1)		15.0	(5.9,24.1)		5.5	(−2.9,13.9)	
6 years (*n* = 1176)	4.8	(2.6,7.0)	<0.001	4.5	(2.2,6.7)	<0.001	1.2	(−0.9,3.3)	0.3
7 years (*n* = 1217)	4.4	(2.3,6.4)	<0.001	4.2	(2.1,6.3)	<0.001	1.3	(−0.7,3.3)	0.2
11 years (*n* = 1219)	2.4	(1.1,3.7)	<0.001	2.1	(0.8,3.5)	0.001	−0.6	(−1.9,0.7)	0.4
15 years (*n* = 1134)	3.8	(2.6,5.0)	<0.001	3.6	(2.4,4.8)	<0.001	1.1	(−0.1,2.4)	0.07
20 years (*n* = 1185)	4.4	(3.3,5.6)	<0.001	4.2	(3.0,5.3)	<0.001	0.4	(−0.8,1.6)	0.5
Height (per cm)									
2 years (*n* = 1106)	1.6	(0.8,2.4)	<0.001	1.8	(0.1,2.7)	<0.001	0.6	(−0.2,1.5)	0.2
4 years (*n* = 1256)	1.2	(0.6,1.8)	<0.001	1.4	(0.8,2.0)	<0.001	0.6	(−0.1,1.3)	0.1
6 years (*n* = 1176)	1.1	(0.5,1.7)	<0.001	1.4	(0.8,2.0)	<0.001	0.3	(−0.4,1.0)	0.5
7 years (*n* = 1217)	1.1	(0.6,1.7)	<0.001	1.3	(0.8,1.9)	<0.001	0.5	(−0.3,1.2)	0.2
11 years (*n* = 1219)	0.9	(0.5,1.3)	<0.001	1.0	(0.6,1.5)	<0.001	0.4	(−0.1,1.0)	0.1
15 years (*n* = 1134)	0.6	(0.2,1.0)	0.005	0.6	(0.2,1.0)	0.003	−0.2	(−0.7,0.4)	0.5
20 years (*n* = 1185)	1.1	(0.6,1.5)	<0.001	1.3	(0.8,1.8)	<0.001	1.0	(−0.1,2.0)	0.08

*Evidence of non-linearity.

**Table 3.** Mean difference (and 95% confidence interval) in left ventricular end diastolic volume (ml) by birthweight and each pair of BMI and height in childhood and adolescence: models adjusted for sex only (model 1); sex and confounders (model 2); sex, confounders and BMI and height at 60–64 years (model 3)

	Adjusted for sex (model 1)			Model 1 + additionally adjusted for childhood and adult social class and (except#) birthweight (model 2)			Model 2 + additionally adjusted for BMI, BMI x sex and height at 60–64 years (model 3)		
	Regression coefficient	(95% CI)	*P*-value	Regression coefficient	(95% CI)	*P*-value	Regression coefficient	(95% CI)	*P*-value
Birthweight# (per kg) (*n* = 1474)	3.4	(0.8,6.1)	0.01	3.5	(0.8,6.1)	0.01	0.8	(−1.8,3.3)	0.6
BMI (per kg/m^2^)									
2 years (*n* = 1180)	1.7	(0.9,2.4)	<0.001	1.6	(0.8,2.4)	<0.001	0.8	(0.1,1.6)	0.04
4 years (*n* = 1330)	1.4	(0.6,2.3)	0.001*	1.3	(0.5,2.2)	0.002*	0.6	(−0.2,1.4)	0.2
Categories									
Lowest fifth	Ref.		0.004	Ref.		0.006			
2	3.1	(−1.1,7.3)		3.0	(−1.2,7.2)				
3	6.1	(2.0,10.3)		6.0	(1.8,10.1)				
4	7.0	(2.7,11.4)		6.8	(2.5, 11.2)				
Highest fifth	6.7	(2.5,11.0)		6.6	(2.3,10.8)				
6 years (*n* = 1240)	2.1	(1.1,3.2)	<0.001	2.1	(1.0,3.1)	<0.001	1.0	(−0.1,2.0)	0.07
7 years (*n* = 1285)	2.2	(1.2,3.1)	<0.001	2.2	(1.2,3.2)	<0.001	1.2	(0.3,2.2)	0.01
11 years (*n* = 1289)	1.2	(0.6,1.9)	<0.001	1.2	(0.6,1.9)	<0.001	0.5	(−0.2,1.1)	0.2
15 years (*n* = 1196)	1.7	(1.1,2.2)	<0.001	1.7	(1.1,2.2)	<0.001	0.9	(0.3,1.5)	0.002
20 years (*n* = 1254)	2.2	(1.7,2.7)	<0.001	2.2	(1.7,2.7)	<0.001	1.1	(0.5,1.6)	<0.001
Height (per cm)									
2 years (*n* = 1180)	0.9	(0.5,1.3)	<0.001	0.9	(0.5,1.3)	<0.001	0.3	(−0.2,0.7)	0.2
4 years (*n* = 1330)	0.6	(0.4,0.9)	<0.001	0.6	(0.3,0.9)	<0.001	0.2	(−0.2,0.5)	0.4
6 years (*n* = 1240)	0.7	(0.5,1.0)	<0.001	0.8	(0.5,1.2)	<0.001	0.2	(−0.2,0.6)	0.3
7 years (*n* = 1285)	0.7	(0.4,0.9)	<0.001	0.7	(0.4,1.0)	<0.001	0.2	(−0.2,0.5)	0.4
11 years (*n* = 1289)	0.5	(0.3,0.7)	<0.001	0.5	(0.3,0.7)	<0.001	0.1	(−0.2,0.4)	0.5
15 years (*n* = 1196)	0.4	(0.2,0.6)	<0.001	0.4	(0.2,0.6)	<0.001	0.0	(−0.2,0.2)	0.8
20 years (*n* = 1254)	0.6	(0.4,0.9)	<0.001	0.6	(0.4,0.9)	<0.001	0.1	(−0.4,0.6)	0.7

*Evidence of non-linearity.

**Table 4.** Mean difference (and 95% confidence interval) in relative wall thickness by birthweight and each pair of BMI and height in childhood and adolescence: models adjusted for sex only (model 1); sex and confounders (model 2); sex, confounders and BMI and height at 60–64 years (model 3)

	Adjusted for sex (model 1)			Model 1 + additionally adjusted for childhood and adult social class and (except#) birthweight (model 2)			Model 2 + additionally adjusted for BMI, BMI x sex and height at 60–64 years (model 3)		
	Regression coefficient	(95% CI)	*P*-value	Regression coefficient	(95% CI)	*P*-value	Regression coefficient	(95% CI)	*P*-value
Birthweight# (per kg) (*n* = 1392)	−0.0060	(−0.0179,0.0007)	0.2	−0.0061	(−0.0156,0.0034)	0.2	−0.0046	(−0.0142,0.0051)	0.4
BMI (per kg/m^2^)									
2 years (*n* = 1106)	−0.0031	(−0.0057,-0.0004)	0.02	−0.0023	(−0.0051,0.0005)	0.1	−0.0022	(−0.0050,0.0006)	0.1
4 years (*n* = 1256)	−0.0007	(−0.0037,0.0023)	0.7	−0.0002	(−0.0033,0.0028)	0.9	−0.0001	(−0.0032,0.0030)	0.9
6 years (*n* = 1176)	0.0009	(−0.0028,0.0045)	0.7	0.0016	(−0.0021,0.0054)	0.4	0.0002	(−0.0036,0.0040)	0.9
7 years (*n* = 1217)	0.0019	(−0.0016,0.0053)	0.3	0.0024	(−0.0011,0.0060)	0.2	0.0008	(−0.0028,0.0043)	0.7
11 years (*n* = 1219)	0.0031	(0.0009,0.0053)	0.006	0.0033	(0.0011,0.0055)	0.004	0.0012	(−0.0012,0.0036)	0.3
15 years (*n* = 1134)	0.0023	(0.0003,0.0042)	0.06	0.0024	(0.0004,0.0044)	0.02	0.0001	(−0.0021,0.0022)	0.96
20 years (*n* = 1185)	0.0009	(−0.0010,0.0028)	0.4	0.0010	(−0.0009,0.0029)	0.3	−0.0011	(−0.0032,0.0011)	0.3
Height (per cm)									
2 years (*n* = 1151)	−0.0005	(−0.0019,0.0008)	0.4	−0.0001	(−0.0015,0.0015)	0.9	0.0003	(−0.0013,0.0018)	0.7
4 years (*n* = 1256)	−0.0007	(−0.0022,0.0008)	0.8	0.0001	(−0.0010,0.0012)	0.8	0.0014	(0.0001,0.0026)	0.04
6 years (*n* = 1176)	−0.0002	(−0.0011,0.0005)	0.8	0.0001	(−0.009,0.0012)	0.8	0.0012	(−0.0002,0.0025)	0.08
7 years (*n* = 1217)	−0.0002	(−0.0011,0.0007)	0.7	−0.0002	(−0.0011,0.0007)	0.7	0.0015	(0.0002,0.0027)	0.02
11 years (*n* = 1219)	−0.0003	(−0.0012,0.0007)	0.6	−0.0005	(−0.0013,0.0003)	0.7	0.0003	(−0.0007,0.0013)	0.5
15 years (*n* = 1134)	−0.0006	(−0.0012,0.0001)	0.1	−0.0005	(−0.0012,0.0002)	0.2	0.0003	(−0.0007,0.0013)	0.5
20 years (*n* = 1185)	−0.0007	(−0.0015,0.0001)	0.06	−0.0007	(−0.0015,0.0001)	0.1	0.0008	(−0.0011,0.0027)	0.4

### BMI and height at each age

BMI and height at every age were positively associated with LVM (Table 2) and LVEDV (Table 3), with suggestion of non-linear relationships with BMI at age 4. There was little confounding by birthweight or life course SEP (Tables 2 and 3). Weaker and less consistent associations were observed with RWT (Table 4). Increasing BMI at age 2 was associated with decreasing RWT, whereas increasing BMI at ages 11 and 15 was associated with increasing RWT (Table 4). The association with BMI at age 2 was somewhat reduced on addition of the confounding variables, with additional investigation suggesting that there was confounding by birthweight.

Associations of early life BMI with LVM and LVEDV were attenuated after adjustment for current BMI and height, although positive associations remained for LVEDV at most ages (Tables 2 and 3). Consistent with this are the associations observed between BMI at each age and LVEDV indexed to either BSA or to height^27^ (Supplementary Data and Supplementary Data). BMI at most ages was, however, also positively (or non-linearly) associated with LVMI. The method of indexation had a much greater influence on associations with height than with BMI, probably because the associations between height and LV structure are a reflection of adult height (Supplementary Data and Supplementary Data). For RWT, the negative association with BMI at 2 years was not affected, but the positive associations with adolescent BMI were considerably reduced after adjustment for current BMI and height (Table 4). Associations with childhood and adolescent height were entirely explained by adult height.

### Conditional BMI and height change

**Figure 1.**
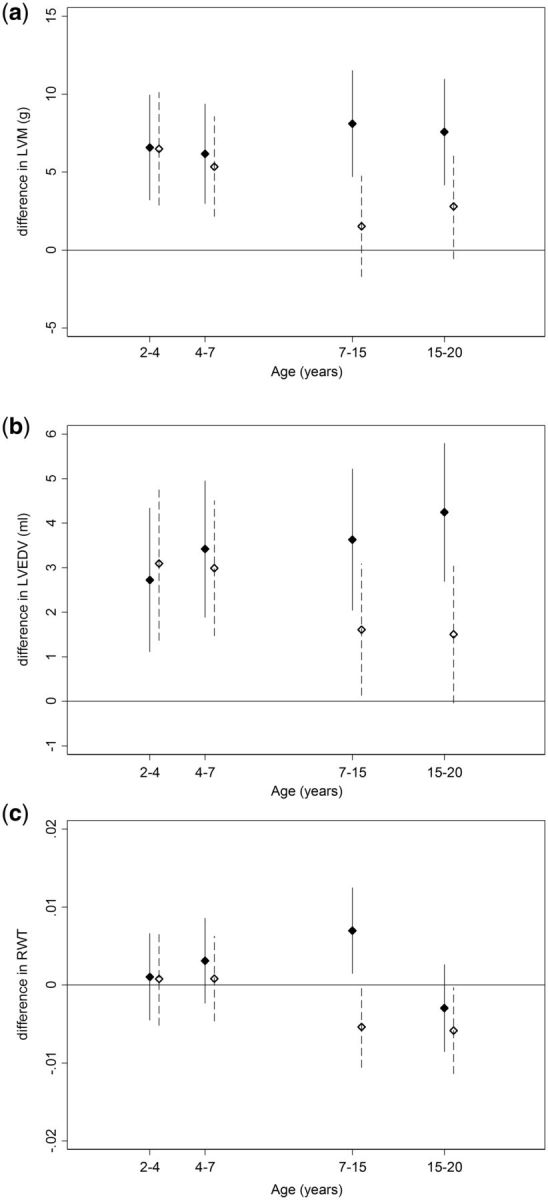
Conditional change (per standard deviation) in BMI (solid) and height (dash) for ages 2–4, 4–7, 7–15 and 15–20 years in relation to a) left ventricular mass (LVM), b) left ventricular end diastolic volume (LVEDV) c) relative wall thickness (RWT). Models adjusted for sex, birthweight, childhood and adult social class. Points are means, vertical bars indicate 95% confidence intervals.

Greater conditional BMI gains during all four age periods considered were associated with higher LVM and LVEDV, with all periods of growth having similar relationships. These associations were only slightly, if at all, attenuated after adjustment for confounders (Figure 1a, b; Supplementary Data, available as Supplementary Data at *IJE* online). Positive associations with LVMI and LVEDVI were substantially weaker for all periods of change, with BMI change from 7–15 years being stronger than earlier periods of change for LVMI, and change from 15–20 years showing the strongest association with LVEDVI when indexed to BSA (Supplementary Data, available as Supplementary Data at *IJE* online). Faster height growth during each time period, particularly in the early periods, was associated with higher LVM and LVEVD before and after adjustment for confounders (Figure 1a, b; Supplementary Data). Height growth was not associated with LVMI or LVEDVI when indexed to BSA (Supplementary Data). Higher BMI gain during the pubertal period of 7–15 years was associated with higher RWT (Figure 1c). Higher systolic BP, diastolic BP and doctor-diagnosed type 2 diabetes were associated with higher LVM, LVEDV and RWT, and associations were slightly attenuated after adjustment for these CV risk factors (Supplementary Data).

### Accumulation of overweight

The more periods during childhood and adolescence that an individual had been overweight, the greater the LVM and LVEDV (Table 5). The association with LVM was attenuated slightly when possible confounders were included and was reduced further towards the null when current overweight and height were added to the model. Associations with LVEDV, although attenuated by adjustment, were not entirely explained (Table 5). These differences were consistent with the findings for outcomes indexed to BSA where an association is seen with LVEDVI but not with LVMI (Supplementary Data, available as Supplementary Data at *IJE* online). Additional analyses showed that CV risk factors also attenuated the association with LVM, but less so for LVEDV.

**Table 5.** Mean difference (and 95% confidence interval) in left ventricular mass (LVM), left ventricular end diastolic volume (LVEDV) and relative wall thickness (RWT) by length of childhood and adolescent overweight: models adjusted for sex only (model 1); sex and confounders (model 2); sex, confounders and overweight and height at 60–64 years (model 3)

	Adjusted for sex (model 1)		Model 1 +additionally adjusted for childhood and adult social class and birthweight (model 2)		Model 2 + additionally adjusted for overweight and height at 60–64 years (model 3)	
	Regression coefficient (95% CI)	*P*-value trend	Regression coefficient (95% CI)	*P*-value trend	Regression coefficient (95% CI)	*P*-value trend
LVM (g) (*n* = 944)						
Number of times overweight						
0 (*n* = 670)	Reference	0.02	Reference	0.07	Reference	0.2
1 (*n* = 201)	3.0 (−5.2,11.1)		1.3 (−6.9,9.5)		1.4 (−6.3,9.2)	
2 (*n* = 61)	11.5 (−2.2,24.9)		8.8 (−4.8,22.4)		4.7 (−8.2,17.5)	
3 (*n* = 12)	31.9 (2.4,61.4)		28.9 (−0.5,58.4)		21.1 (−6.8,48.9)	
LVEDV (ml) (*n* = 995)						
Number of times overweight						
0 (*n* = 703)	Reference	< 0.001	Reference	< 0.001	Reference	0.001
1 (*n* = 212)	4.5 (0.6,8.3)		4.3 (0.4,8.2)		4.2 (0.4,7.9)	
2 (*n* = 67)	10.0 (3.7,16.2)		9.7 (3.4,16.0)		8.5 (2.5,14.6)	
3 (*n* = 13)	12.3 (−1.5,26.0)		11.8 (−1.9,25.6)		10.2 (−3.1,23.4)	
RWT (*n* = 944)						
Number of times overweight						
0 (*n* = 670)	Reference	0.3	Reference	0.4	Reference	0.2
1 (*n* = 201)	−0.007 (−0.020,0.007)		−0.006 (−0.020,0.007)		−0.008 (−0.022,0.006)	
2 (*n* = 61)	−0.007 (−0.029,0.016)		−0.006 (−0.029,0.016)		−0.010 (−0.033,0.012)	
3 (*n* = 12)	−0.010 (−0.058,0.039)		−0.009 (−0.057,0.040)		−0.015 (−0.063,0.034)	

### LV remodelling

Birthweight was not associated with LV remodelling category. Higher BMI from 6 years onwards was associated with greater odds of concentric LVH compared with normal (Supplementary Data, available as Supplementary Data at *IJE* online). A similar pattern of association, although much weaker, was observed for eccentric LVH whereas no associations with BMI were apparent for concentric remodelling. Change in BMI from ages 7 to 15 was most strongly associated with LV remodelling, and in particular concentric LVH (Supplementary Data, available as Supplementary Data at *IJE* online).

## Discussion

In this nationally representative cohort study with over 60 years of follow-up, birthweight was positively associated with LVM and LVEDV at age 60–64, with evidence of a levelling off or decline in the association at higher birthweights for LVM. These associations were explained by adult height. Associations between early life body size and LVM and LVEDV were broadly similar; higher BMI and taller height at all ages, greater changes in BMI and height and a greater accumulation of overweight were associated with higher values. Some of these associations, more notably for LVM, were mediated through adult BMI. All associations with height in childhood were accounted for by adult height. There were less consistent and weaker associations with RWT.

A major strength of the study is the availability of multiple measurements of height and weight throughout childhood and adolescence, together with the standardized measures of cardiac structure using echocardiography in a relatively large sample of people in early old age. This provides a unique opportunity to investigate the relative strengths of associations with BMI at different ages and changes in body size.

The NSHD is representative of the British-born population in 1946, and therefore we are unable to study ethnic differences or to generalize our findings beyond the White British population of similar age. Those included in the analysis were taller and had lower BMI at 60–64 than those who provided data at 60–64 but were not included in the analysis, largely those who had a home visit instead of a clinic visit. Previous analysis shows that those attending clinic were healthier than those who had a home visit, and thus the analysis sample is likely to be healthier than the general population.^23^ Given the healthier characteristics of the sample and the low levels of childhood overweight and obesity in NSHD, associations with BMI might, if anything, be underestimated in our analyses. BMI was the only measure of adiposity available in childhood; nevertheless. although it does not measure fat mass directly, it has been shown to be a reasonable measure of adiposity in childhood.^26^(26)

Modelling growth in relation to a subsequent health outcome is challenging.^27–29^ In the NSHD, heights and weights were measured at fixed ages and were relatively widely spaced. We thus used a conditional change approach, as there is little or nothing to be gained from more complex modelling of the growth trajectory which is more beneficial with age-heterogeneous measurements.^30^ The conditional change model can be interpreted as the change in body size above or below that expected given earlier BMI or height, and is useful in identifying accelerated or restricted growth.^29^ LV size is known to scale in proportion to body size but the appropriate mode of indexation remains contentious; indexing LVM to BSA underestimates LVH among those who are obese.^31^^,^^32^ In our primary analyses, we therefore chose not to index LV mass but to include height and BMI as covariates in models, thereby avoiding potential problems with age-related differences in optimal scaling index. However, sensitivity analyses using indexed values supported the conclusions of the primary analyses.

Our findings for birthweight are in contrast to previous studies, which have generally found no association between birthweight and LVM. Our study contains a larger sample size and thus has greater statistical power than most previous studies, particularly to detect a possible non-linear association. That the birthweight association was largely explained by adult height suggests that associations reflect allometric scaling of LV size to body size.^33^ Prematurity may be a more important risk factor for LVH than birthweight, as a recent study has shown increased LVM and remodelling of the hearts of adults who were born preterm (gestational age 30.3 ± 2.5 weeks; birthweight 1297 ± 287 g).^13^ Gestational age was not available in the NSHD and thus we could not investigate this. However, in 1946, few premature babies would have survived^34^ and thus the observed association with birthweight is unlikely to represent, or be confounded by, gestational age. Younger cohorts are thus required, to disentangle the effects of gestational age and birthweight. Previous studies have observed a negative association between higher weight in early postnatal life and lower LVM in adulthood^11^^,^^12^ rather than lower RWT, as in the current study. The association between high BMI at age 2 and lower RWT is a result of the stronger positive relationship between BMI at that age and LVEDV compared with LVM. Infant weight gain may have a different impact on overall LV structure compared with later childhood and adolescent BMI change.

For LVM and particularly for LVEDV, our evidence suggests that high BMI from early childhood onwards has consequences for LV structure in later life. Consistent with our findings, those from the Bogalusa Heart Study suggested that higher childhood BMI was associated with higher LVMI in young adulthood.^14^ However, that sample was age-heterogeneous, meaning that the measure of childhood BMI used was recorded at ages varying from 4 to 17 years. Consequently, it was not possible to determine whether BMI at all ages was similarly associated with LVMI. For LVM, LVEDV and their indexed values, we showed consistent associations with BMI across childhood and adolescence. More rapid changes in BMI at all ages were positively associated with LVM and LVEDV without evidence of there being a sensitive period in which gain in BMI is particularly detrimental. The most recent of two reports on LV remodelling in the Bogalusa Heart Study showed that a greater cumulative exposure to higher BMI (using the area under the curve for individual study members) was related to higher LVMI and greater odds of LVH.^16^ This is consistent with our findings, namely that the greater the duration of overweight in childhood and adolescence, the higher the LVM and LVEDV. For LVEDV this was seen even among those currently not overweight and was independent of CV risk factors. For LVM, the association was largely explained by adult height and BMI. The fact that associations were observed between higher BMI in childhood and higher LVM indexed to BSA might suggest mediation through adult adiposity rather than the associations simply reflecting general adult body size.

Studies in childhood and adolescence show that high BMI and overweight are associated with higher LVM.^6^ A systematic review of cross-sectional studies in children found that those who were obese had an LVM on average 19.12 (12.66,25.59)g greater than children of normal weight.^35^ The increased LVEDV in those with accumulated exposure to overweight is interpretable as an indicator of elevated preload, and this is consistent with evidence of enhanced recruitment of preload reserve in obesity.^36^ Our findings for LVM are consistent with studies which suggest that some reversal of elevated LVM is possible with weight loss.^37^^,^^38^ However, it is uncertain whether complete reversibility can be achieved, particularly following more severe overweight or obesity. We showed only weak evidence that associations may explained by adult BP, heart rate and type 2 diabetes. In this cohort, faster rises in midlife BP, as well as contemporaneous BP, are associated with detriments to LV structure and diastolic function.^39^^,^^40^ Childhood overweight is associated with increased levels of CV risk factors such as BP, total cholesterol and glucose in childhood.^35^ Therefore cumulative exposure to higher BP and other CV risk factors, which the current measures do not reflect, may be one pathway through which accumulation of high BMI from childhood is associated with high LVM and LVEDV in older age.

For the most part, changes in LVM and LVEDV linked with BMI occurred without much effect on RWT. The exception was the stronger effect of pubertal BMI gain and the similar finding that rapid BMI gain from ages 7 to 15 was most strongly related to concentric hypertrophy characterized by an increased RWT and increased LVM. Few studies have investigated the associations of childhood body size with RWT or LVEDV. A small Australian study with echocardiography in young adulthood^41^ showed that childhood overweight was not related with RWT.

Tracking of body size from childhood to adulthood means that it is difficult to disentangle the impact of early life body size from current body size.^42^ Adjustment for adult body size in the analyses makes strong assumptions^43^ which, if they do not hold, may bias the findings. Height growth in childhood does not appear to have an association with later life LV structure over and above that of adult height, and thus is likely to reflect LV scaling to normal somatic growth. Given that in the NSHD cohort fewer children were overweight or obese compared with more contemporary cohorts,^44^ we might expect associations between childhood BMI and accumulation of overweight with LV structure to be stronger, and of greater public health importance in later-born generations. Even if childhood and adolescent overweight or obesity does not have a large direct effect on later life LV structure, there is an indirect effect through the tracking of BMI from childhood through to adulthood.

### Conclusions

High BMI and overweight across childhood and adolescence and accumulation of overweight across the life course was associated with greater LVM and LVEDV. These associations, particularly those with LVM, were partially mediated by adult body size. These findings are of particular public health relevance given the increasing levels of overweight in contemporary cohorts of children and emphasises the need for effective interventions to reduce childhood overweight.

## Funding

This work is supported by the UK Medical Research Council which provides core funding for the MRC National Survey of Health and Development and R.H. and D.K. [MC_UU_12019/1,MC_UU_12019/2]. A.D.H. and J.E.D. received support from the National Institute for Health Research University College London Hospitals Biomedical Research Centre.

## Supplementary Material

Supplementary Data

Supplementary Data
