# Supplementary material for: Birthweight, childhood growth and left ventricular structure at age 60–64 years in a British birth cohort study
Source: Int J Epidemiol. 2016 Jul 13;45(4):1091–102. doi: 10.1093/ije/dyw150 (PMC5841632; doi:10.1093/ije/dyw150)
Supplement: Supplementary Data [file dyw150_supplementary_data.zip › ije-2015-10-1444-File006.docx]

Supplementary table 1: Mean difference (and 95% confidence interval) in left ventricular mass indexed to a) body surface area (g/m^2^) and b) height^2.7^ by birth weight and each pair of BMI and height in childhood and adolescence: models adjusted for sex only; sex and confounders; sex, confounders and BMI and height at 60-64 years.

|  | Adjusted for sex | | | Additionally adjusted for childhood and adult social class  and (except ^¶^) birth weight | | |
| --- | --- | --- | --- | --- | --- | --- |
|  | Regression coefficient | (95% CI) | p-value | Regression coefficient | (95% CI) | p-value |
| **a) Indexed to BSA(g/m^2^)** | | | | | | |
| **Birth weight** (per kg) (n=1392)  Categories  <2.5kg  2.5-3.0kg  3.0-3.5kg  3.5-4.0kg  4.0kg-4.5kg  >=4.5kg | 0.6  1.6  -0.8  Ref.  2.8  -0.5  -1.4 | (-2.2,3.3)  (-2.3,5.5)  (-9.6,8.1)  (-1.2,6.8)  (-6.4,5.4)  (-11.1,8.3) | 0.7*  0.6 | 0.6  1.5  -2.0  Ref.  2.7  -0.9  -1.5 | (-2.2,3.3)  (-2.4,5.4)  (-10.8,6.9)  (-1.2,6.7)  (-6.8,5.0)  (-11.2,8.1) | 0.7*  0.6 |
| **BMI** (per kg/m^2^) |  |  |  |  |  |  |
| 2 years (n=1106) | 0.2 | (-0.6,1.0) | 0.6 | 0.3 | (-0.5,1.2) | 0.4 |
| 4 years (n=1256)  Categories  Lowest fifth  2  3  4  Highest fifth | 1.0  Ref.  1.2  2.2  6.8  4.1 | (0.1,1.8)  (-3.1,5.5)  (-2.0,6.4)  (2.3,11.2)  (-0.2,8.4) | 0.03*  0.03 | 0.9  Ref.  0.6  1.9  6.3  3.7 | (0.0,1.8)  (-3.7,4.9)  (-2.4,6.1)  (1.8,10.8)  (-0.7,8.1) | 0.04  0.04 |
| 6 years (n=1176)  Categories  Lowest fifth  2  3  4  Highest fifth | 1.5  Ref.  6.8  5.1  7.7  6.8 | (0.4,2.5)  (2.3,11.3)  (0.8,9.5)  (3.1,12.2)  (2.3,11.3) | 0.006*  0.006 | 1.4  Ref.  6.2  4.8  7.1  6.4 | (0.3,2.5)  (1.7,10.8)  (0.4,9.2)  (2.4,11.7)  (1.9,11.0) | 0.01  0.02 |
| 7 years (n=1217) | 1.5 | (0.5,2.5) | 0.005 | 1.5 | (0.4,2.5) | 0.006 |
| 11 years (n=1219) | 0.7 | (0.1,1.4) | 0.03 | 0.6 | (0.0,1.3) | 0.05 |
| 15 years (n=1134) | 1.4 | (0.9,2.0) | <0.001 | 1.4 | (0.8,1.9) | <0.001 |
| 20 years (n=1185) | 1.2 | (0.7,1.8) | <0.001 | 1.1 | (0.5,1.6) | <0.001 |
| **Height** (per cm) |  |  |  |  |  |  |
| 2 years (n=1106) | 0.1 | (-0.3,0.5) | 0.6 | 0.3 | (-0.5,0.7) | 0.4 |
| 4 years (n=1256) | -0.1 | (-0.3,0.2) | 0.8 | 0.1 | (-0.3,0.4) | 0.7 |
| 6 years (n=1176) | -0.1 | (-0.4,0.2) | 0.3 | 0.0 | (-0.3,0.3) | 0.95 |
| 7 years (n=1217) | -0.1 | (-0.4,0.2) | 0.4 | 0.0 | (-0.3,0.3) | 0.9 |
| 11 years (n=1219) | -0.1 | (-0.3,0.2) | 0.5 | 0.0 | (-0.2,0.2) | 0.99 |
| 15 years (n=1134) | -0.2 | (-0.4,0.0) | 0.09 | -0.1 | (-0.3,0.1) | 0.1 |
| 20 years (n=1185) | -0.2 | (-0.4,0.0) | 0.1 | -0.1 | (-0.3,0.1) | 0.4 |
| **b) Indexed to height^2.7^(g/m^2.7^)** | | | | | | |
| **Birth weight** (per kg) (n=1392)  Categories  <2.5kg  2.5-3.0kg  3.0-3.5kg  3.5-4.0kg  4.0kg-4.5kg  >=4.5kg | -0.5  0.4  0.5  Ref.  0.6  -1.4  -2.2 | (-1.9,0.9)  (-1.7,2.4)  (-4.1,5.0)  (-1.4,2.7)  (-4.5,1.6)  (-7.3,2.8) | 0.5*  0.7 | -0.5  0.3  -0.3  Ref  0.6  -1.7  -2.3 | (-1.8,1.0)  (-1.7,2.3)  (-4.8,4.2)  (-1.5,2.6)  (-4.7,1.3)  (-7.3,2.7) | 0.5*  0.6 |
| **BMI** (per kg/m^2^) |  |  |  |  |  |  |
| 2 years (n=1106) | -0.1 | (-0.5,0.3) | 0.8 | 0.1 | (-0.3,0.5) | 0.7 |
| 4 years (n=1256)  Categories  Lowest fifth  2  3  4  Highest fifth | 0.5  Ref.  1.2  1.6  4.0  2.4 | (0.1,1.0)  (-1.1,3.4)  (-0.7,3.7)  (1.7,6.3)  (0.2,4.6) | 0.02*  0.01 | 0.5  Ref.  0.9  1.4  3.8  2.2 | (0.1,0.9)  (-1.3,3.1)  (-0.8,3.6)  (1.4,6.1)  (-0.1,4.5) | 0.03  0.02 |
| 6 years (n=1176)  Categories  Lowest fifth  2  3  4  Highest fifth | 1.1  Ref.  3.6  3.4  4.9  4.7 | (0.5,1.6)  (1.3,6.0)  (1.2,5.7)  (2.6,7.3)  (2.4,7.0) | <0.001*  <0.001 | 1.1  Ref.  3.3  3.2  4.6  4.6 | (0.5,1.6)  (1.0,5.7)  (1.0,5.4)  (2.3,7.0)  (2.3,7.0) | <0.001  <0.001 |
| 7 years (n=1217) | 1.1 | (0.6,1.7) | <0.001 | 1.2 | (0.6,1.7) | <0.001 |
| 11 years (n=1219) | 0.9 | (0.6,1.2) | <0.001 | 0.9 | (0.5,1.2) | <0.001 |
| 15 years (n=1134) | 1.2 | (0.9,1.5) | <0.001 | 1.2 | (0.9,1.5) | <0.001 |
| 20 years (n=1185) | 1.1 | (0.8,1.4) | <0.001 | 1.0 | (0.7,1.3) | <0.001 |
| **Height** (per cm) |  |  |  |  |  |  |
| 2 years (n=1106) | -0.1 | (-0.3,0.1) | 0.2 | 0.1 | (-0.3,0.5) | 0.7 |
| 4 years (n=1256) | -0.3 | (-0.4,-0.1) | 0.001 | -0.2 | (-0.3,0.0) | 0.03 |
| 6 years (n=1176) | -0.3 | (-0.4,-0.1) | <0.001 | -0.2 | (-0.3,0.0) | 0.01 |
| 7 years (n=1217) | -0.3 | (-0.4,-0.1) | <0.001 | -0.2 | (-0.3,-0.1) | 0.006 |
| 11 years (n=1219) | -0.2 | (-0.3,-0.1) | <0.001 | -0.2 | (-0.3,-0.1) | 0.003 |
| 15 years (n=1134) | -0.3 | (-0.4,-0.2) | <0.001 | -0.2 | (-0.3,-0.1) | <0.001 |
| 20 years (n=1185) | -0.4 | (-0.5,-0.2) | <0.001 | -0.3 | (-0.4,-0.2) | <0.001 |

* evidence of non-linearity

Supplementary table 2: Mean difference (and 95% confidence interval) in left ventricular end diastolic volume indexed to a) body surface area (ml/m^2^) and b) height^2.7^ (ml/m^2.7^) by birth weight and each pair of BMI and height in childhood and adolescence: models adjusted for sex only; sex and confounders; sex, confounders and BMI and height at 60-64 years.

|  | Adjusted for sex | | | | Additionally adjusted for childhood and adult social class  and (except ^¶^) birth weight | | |
| --- | --- | --- | --- | --- | --- | --- | --- |
|  | Regression coefficient | (95% CI) | p-value | | Regression coefficient | (95% CI) | p-value |
| **a) Indexed to BSA(ml/m^2^)** | | | | | | | |
| **Birth weight**^¶^ (per kg) (n=1474) | 0.13 | (-1.2,1.4) | 0.8 | | 0.12 | (-1.2,1.4) | 0.9 |
| **BMI** (per kg/m^2^) |  |  |  | |  |  |  |
| 2 years (n=1180) | 0.35 | (-0.02,0.72) | 0.07 | | 0.40 | (0.01,0.80) | 0.05 |
| 4 years (n=1330) | 0.28 | (-0.12,0.69) | 0.2 | | 0.28 | (-0.13,0.70) | 0.2 |
| 6 years (n=1240) | 0.54 | (0.03,1.04) | 0.04 | | 0.57 | (0.05,1.1) | 0.03 |
| 7 years (n=1285) | 0.69 | (0.22,1.16) | 0.004 | | 0.75 | (0.27,1.23) | 0.002 |
| 11 years (n=1289) | 0.36 | (0.05,0.67) | 0.02 | | 0.38 | (0.07,0.69) | 0.02 |
| 15 years (n=1196) | 0.51 | (0.24,0.79) | <0.001 | | 0.53 | (0.25,0.80) | <0.001 |
| 20 years (n=1254) | 0.52 | (0.26,0.77) | <0.001 | | 0.54 | (0.29,0.80) | <0.001 |
| **Height** (per cm) |  |  |  | |  |  |  |
| 2 years (n=1180) | 0.08 | (-0.12,0.72) | 0.5 | | 0.10 | (-0.10,0.30) | 0.3 |
| 4 years (n=1330) | -0.04 | (-0.18,0.10) | 0.6 | | -0.04 | (-0.18,0.11) | 0.6 |
| 6 years (n=1240) | 0.00 | (-0.14,0.13) | 0.97 | | 0.01 | (-0.13,0.16) | 0.9 |
| 7 years (n=1285) | -0.03 | (-0.16,0.10) | 0.6 | | -0.01 | (-0.15,0.12) | 0.8 |
| 11 years (n=1289) | -0.02 | (-0.13,0.08) | 0.6 | | -0.02 | (-0.12,0.09) | 0.7 |
| 15 years (n=1196) | -0.04 | (-0.13,0.05) | 0.4 | | -0.04 | (-0.14,0.05) | 0.4 |
| 20 years (n=1254) | -0.07 | (-0.18,0.03) | 0.2 | | -0.08 | (-0.19,0.03) | 0.2 |
| **b) Indexed to height^2.7^(ml/m^2.7^)** | | | | | | | |
| **Birth weight**^¶^ (per kg) (n=1474) | -0.40 | (-1.04,0.25) | | 0.2 | -0.40 | (-1.04,0.25) | 0.2 |
| **BMI** (per kg/m^2^) |  |  | |  |  |  |  |
| 2 years (n=1180) | 0.06 | (-0.13,0.24) | | 0.5 | 0.13 | (-0.07,0.32) | 0.2 |
| 4 years (n=1330) | 0.14 | (-0.07,0.34) | | 0.2 | 0.14 | (-0.07,0.35) | 0.2 |
| 6 years (n=1240) | 0.44 | (0.19,0.70) | | 0.001 | 0.47 | (0.21,0.73) | <0.001 |
| 7 years (n=1285) | 0.53 | (0.30,0.78) | | <0.001 | 0.58 | (0.34,0.81) | <0.001 |
| 11 years (n=1289) | 0.46 | (0.31,0.62) | | <0.001 | 0.48 | (0.33,0.63) | <0.001 |
| 15 years (n=1196) | 0.53 | (0.40,0.66) | | <0.001 | 0.54 | (0.41,0.68) | <0.001 |
| 20 years (n=1254) | 0.52 | (0.40,0.64) | | <0.001 | 0.53 | (0.40,0.65) | <0.001 |
| **Height** (per cm) |  |  | |  |  |  |  |
| 2 years (n=1180) | -0.09 | (-0.18,0.01) | | 0.07 | -0.04 | (-0.14,0.06) | 0.4 |
| 4 years (n=1330) | -0.15 | (-0.22,-0.08) | | <0.001 | -0.13 | (-0.20,-0.06) | <0.001 |
| 6 years (n=1240) | -0.12 | (-0.19,-0.06) | | <0.001 | -0.10 | (-0.17,-0.03) | 0.006 |
| 7 years (n=1285) | -0.13 | (-0.20,-0.07) | | <0.001 | -0.11 | (-0.18,-0.05) | 0.001 |
| 11 years (n=1289) | -0.12 | (-0.17,-0.07) | | <0.001 | -0.11 | (-0.16,-0.06) | <0.001 |
| 15 years (n=1196) | -0.12 | (-0.16,-0.07) | | <0.001 | -0.11 | (-0.16,-0.07) | <0.001 |
| 20 years (n=1254) | -0.18 | (-0.23,-0.13) | | <0.001 | -0.17 | (-0.23,-0.12) | <0.001 |

Supplementary table 3: Mean difference (and 95% confidence interval) in left ventricular mass (LVM), left ventricular end diastolic volume (LVEDV) and relative wall thickness (RWT) for a 1 standard deviation conditional change in BMI and height: models adjusted for sex only, sex and confounders, and sex, confounders and CV risk factors at 60-64 years

|  | Adjusted for sex | | Additionally adjusted for childhood and adult social class and birth weight | | Additionally adjusted for BP, HR and diabetes at 60-64y | |
| --- | --- | --- | --- | --- | --- | --- |
|  | Regression coefficient  (95% CI) | p-value | Regression coefficient  (95% CI) | p-value | Regression coefficient  (95% CI) | p-value |
| **LVM** |  |  |  |  |  |  |
| Change 2-4y (n=967)  BMI (per SD)  Height (per SD) | 7.1 (3.6,10.6)  6.5 (2.9,10.1) | <0.001  <0.001 | 6.7 (3.2,10.25)  7.2 (3.5,10.94) | <0.001  <0.001 | 6.8 (3.4,10.2)  7.5 (3.9,11.1) | <0.001  <0.001 |
| Change 4-7 (n=1018)  BMI (per SD)  Height (per SD) | 6.4 (3.1,9.7)  5.0 (1.6,8.4) | <0.001  0.004 | 6.3 (3.0,9.6)  4.9 (1.5,8.4) | <0.001  0.005 | 5.8 (2.6,9.0)  4.2 (0.9,7.5) | <0.001  0.01 |
| Change 7-15y (n=944)  BMI (per SD)  Height (per SD) | 8.3 (4.7,11.9)  1.7 (-1.7,5.1) | <0.001  0.3 | 7.7 (4.1,11.2)  1.3 (-2.1,4.8) | <0.001  0.4 | 6.6 (3.1,10.1)  0.7 (-2.6,4.1) | <0.001  0.7 |
| Change 15-20y (n=919)  BMI (per SD)  Height (per SD) | 7.9 (4.5,11.4)  2.4 (-1.1,5.9) | <0.001  0.2 | 7.0 (3.5,10.5)  2.4 (-1.1,5.9) | <0.001  0.2 | 5.8 (2.3,9.2)  2.7 (-0.7,6.2) | 0.001  0.1 |
| **LVEDV** |  |  |  |  |  |  |
| Change 2-4y (n=1027)  BMI (per SD)  Height (per SD) | 3.2 (1.5,4.8)  3.4 (1.7,5.1) | <0.001  <0.001 | 3.0 (1.3,4.7)  3.3 (1.5,5.1) | 0.001  <0.001 | 3.0 (1.3,4.6)  3.3 (1.5,5.0) | <0.001  <0.001 |
| Change 4-7 (n=1074)  BMI (per SD)  Height (per SD) | 3.5 (1.9,5.0)  2.7 (1.1,4.3) | <0.001  0.001 | 3.4 (1.8,5.0)  2.7 (1.0,4.3) | <0.001  0.001 | 3.2 (1.7,4.8)  2.5 (0.9,4.1) | <0.001  0.002 |
| Change 7-15y (n=994)  BMI (per SD)  Height (per SD) | 3.3 (1.7,5.0)  1.8 (0.2,3.3) | <0.001  0.03 | 3.3 (1.6,4.9)  1.7 (0.1,3.2) | <0.001  0.04 | 2.9 (1.3,4.5)  1.3 (-0.3,2.8) | <0.001  0.1 |
| Change 15-20y (n=970)  BMI (per SD)  Height (per SD) | 4.3 (2.7,5.8)  1.8 (0.7,3.3) | <0.001  0.03 | 4.3 (2.7,5.9)  1.6 (-0.1,3.1) | <0.001  0.06 | 4.0 (2.4,5.5)  1.7 (0.1,3.2) | <0.001  0.04 |
| **RWT** |  |  |  |  |  |  |
| Change 2-4y (n=967)  BMI (per SD)  Height (per SD) | 0.000 (-0.006,0.006)  0.000 (-0.006,0.006) | 0.97  0.9 | -0.001 (-0.0050,0.0068)  -0.002 (-0.0044,0.0080) | 0.8  0.6 | 0.001 (-0.005,0.007)  0.002 (-0.004,0.008) | 0.7  0.5 |
| Change 4-7 (n=1018)  BMI (per SD)  Height (per SD) | 0.002 (-0.017,0.005)  0.001 (-0.005,0.006) | 0.5  0.9 | 0.003 (-0.003,0.009)  0.001 (-0.005,0.007) | 0.3  0.7 | 0.003 (-0.002,0.009)  0.000 (-0.006,0.006) | 0.3  0.9 |
| Change 7-15y (n=944)  BMI (per SD)  Height (per SD) | 0.007 (0.001,0.013)  -0.006 (-0.011,-0.001) | 0.02  0.04 | 0.007 (0.001,0.013)  -0.006 (-0.011,0.000) | 0.02  0.04 | 0.007 (0.001,0.012)  -0.006 (-0.011,0.000) | 0.03  0.05 |
| Change 15-20y (n=919)  BMI (per SD)  Height (per SD) | -0.004 (-0.010,0.002)  -0.007 (-0.013,-0.001) | 0.2  0.03 | -0.004 (-0.010,0.002)  -0.006 (-0.012,-0.001) | 0.2  0.03 | -0.005 (-0.011,0.001)  -0.007 (-0.012,-0.001) | 0.09  0.03 |

Abbreviations: BMI, body mass index; HR, heart rate; BP, blood pressure. Note that the sample sizes are based on those with complete information on all confounding and CV risk factor information and are therefore smaller than the samples on which Figure 1 is based and so coefficients will not be exactly.

Supplementary table 4: Mean difference (and 95% confidence interval) in left ventricular mass (LVMI), left ventricular end diastolic volume (LVEDVI) indexed to body surface area for a 1 standard deviation conditional change in BMI and height: models adjusted for sex only, sex and confounders, and sex, confounders and CV risk factors at 60-64 years

|  | Adjusted for sex | | Additionally adjusted for childhood and adult social class and birth weight | | Additionally adjusted for BP, HR and diabetes at 60-64y | |
| --- | --- | --- | --- | --- | --- | --- |
|  | Regression coefficient  (95% CI) | p-value | Regression coefficient  (95% CI) | p-value | Regression coefficient  (95% CI) | p-value |
| **LVMI** |  |  |  |  |  |  |
| Change 2-4y (n=967)  BMI (per SD)  Height (per SD) | 1.7 (0.0,3.4)  0.4 (-1.3,2.1) | 0.05  0.6 | 1.7 (0.0,3.4)  1.0 (-0.8,2.8) | 0.05  0.3 | 1.7 (0.1,3.4)  1.1 (-0.7,2.8) | 0.04  0.2 |
| Change 4-7 (n=1018)  BMI (per SD)  Height (per SD) | 1.6 (0.0,3.2)  -0.3 (-1.9,1.4) | 0.05  0.8 | 1.7 (0.1,3.3)  -0.2 (-1.9,1.5) | 0.04  0.8 | 1.5 (-0.1,3.0)  -0.5 (-2.1,1.1) | 0.07  0.6 |
| Change 7-15y (n=944)  BMI (per SD)  Height (per SD) | 2.8 (1.1,4.5)  -0.8 (-2.4,0.9) | 0.002  0.4 | 2.6 (0.8,4.3)  -0.9 (-2.5,0.8) | 0.004  0.3 | 2.1 (0.4,3.8)  -1.1 (-2.8,0.5) | 0.02  0.2 |
| Change 15-20y (n=919)  BMI (per SD)  Height (per SD) | 2.0 (0.4,3.7)  -1.1 (-2.8,0.6) | 0.02  0.2 | 1.6 (-0.1,3.3)  -1.0 (-2.7,0.7) | 0.07  0.3 | 1.1 (-0.5,2.8)  -0.8 (-2.5,0.8) | 0.2  0.3 |
| **LVEDVI** |  |  |  |  |  |  |
| Change 2-4y (n=1027)  BMI (per SD)  Height (per SD) | 0.7 (-0.2,1.5)  0.2 (-0.7,1.0) | 0.1  0.7 | 0.7 (-0.2,1.5)  0.2 (-0.6,1.1) | 0.1  0.6 | 0.7 (-0.2,1.5)  0.2 (-0.7,1.0) | 0.1  0.7 |
| Change 4-7 (n=1074)  BMI (per SD)  Height (per SD) | 0.8 (0.1,1.6)  -0.2 (-1.0,0.6) | 0.03  0.6 | 0.9 (0.1,1.7)  -0.2 (-1.0,0.6) | 0.02  0.7 | 0.8 (0.1,1.6)  -0.2 (-1.0,0.6) | 0.03  0.6 |
| Change 7-15y (n=994)  BMI (per SD)  Height (per SD) | 0.7 (-0.1,1.5)  0.0 (-0.8,0.7) | 0.08  0.96 | 0.7 (-0.1,1.5)  0.0 (-0.8,0.7) | 0.08  0.98 | 0.6 (-0.2,1.4)  -0.2 (-0.9,0.5) | 0.1  0.96 |
| Change 15-20y (n=970)  BMI (per SD)  Height (per SD) | 1.2 (0.4,2.0)  -0.4 (-1.2,0.4) | 0.004  0.3 | 1.2 (0.4,2.0)  -0.4 (-1.2,0.4) | 0.004  0.3 | 1.1 (0.3,1.8)  -0.4 (-1.2,0.4) | 0.007  0.3 |

Abbreviations: BMI, body mass index; HR, heart rate; BP, blood pressure.

Supplementary table 5: Mean difference (and 95% confidence interval) in left ventricular mass index (LVMI) and left ventricular end diastolic volume index (LVEDVI) by length of childhood and adolescent overweight: models adjusted for sex only; sex and confounders.

|  | Adjusted for sex | | Additionally adjusted for childhood and adult social class and birth weight | |
| --- | --- | --- | --- | --- |
|  | Regression coefficient (95% CI) | p-value  trend | Regression coefficient (95% CI) | p-value  trend |
| **LVMI** BSA (g/m^2^)  (n=944) |  |  |  |  |
| Number of times overweight  0 (n=670)  1 (n=201)  2 (n=61)  3 (n=12) | Reference  1.2 (-2.7,5.1)  2.7 (-3.8,9.2)  10.7 (-3.4,24.9) | 0.1 | Reference  0.8 (-3.1,4.7)  2.0 (-4.9,8.5)  10.1 (-4.1,24.2) | 0.2 |
| **LVEDVI** (ml/m^2^)  (n=995) |  |  |  |  |
| Number of times overweight  0 (n=703)  1 (n=212)  2 (n=67)  3 (n=13) | Reference  1.8 (0.0,3.7)  3.5 (0.4,6.6)  3.7 (-3.0,10.4) | 0.004 | Reference  2.0 (0.1,3.9)  3.7 (0.6,6.8)  3.9 (-2.8,10.6) | 0.003 |

Supplementary table 6: Odds ratios (OR) for geometric remodelling categories compared with normal left ventricular structure by birth weight and each pair of BMI and height in childhood and adolescence: models adjusted for sex

|  |  | Eccentric hypertrophy | | Concentric hypertrophy | | Concentric remodelling | |
| --- | --- | --- | --- | --- | --- | --- | --- |
|  | n | OR (95% CI) | p-value | OR (95% CI) | p-value | OR (95% CI) | p-value |
| **Birth weight** (per kg) | 1475 | 1.07 (0.78,1.47) | 0.7 | 0.91 (0.67,1.22) | 0.5 | 0.81 (0.63,1.04) | 0.1 |
| **BMI** (per kg/m^2^) |  |  |  |  |  |  |  |
| 2 years | 1106 | 1.03 (0.93,1.14) | 0.6 | 1.00 (0.90,1.10) | 0.95 | 0.97 (0.89,1.05) | 0.4 |
| 4 years | 1256 | 1.07 (0.96,1.19) | 0.2 | 1.04 (0.93,1.15) | 0.5 | 0.99 (0.91,1.08) | 0.8 |
| 6 years | 1176 | 1.08 (0.95,1.23) | 0.3 | 1.15 (1.01,1.30) | 0.03 | 1.00 (0.90,1.12) | 0.95 |
| 7 years | 1217 | 1.08 (0.96,1.22) | 0.2 | 1.12 (1.00,1.26) | 0.06 | 1.00 (0.91,1.11) | 0.96 |
| 11 years | 1219 | 1.06 (0.99,1.15) | 0.1 | 1.12 (1.04,1.20) | 0.003 | 1.04 (0.97,1.11) | 0.3 |
| 15 years | 1134 | 1.07 (1.00,1.15) | 0.07 | 1.13 (1.06,1.21) | <0.001 | 1.01 (0.95,1.08) | 0.7 |
| 20 years | 1185 | 1.04 (0.96,1.11) | 0.3 | 1.12 (1.05,1.19) | <0.001 | 1.00 (0.94,1.06) | 0.8 |
| **Height** (per cm) |  |  |  |  |  |  |  |
| 2 years | 1106 | 1.03 (0.98,1.08) | 0.6 | 1.04 (0.99,1.09) | 0.2 | 1.01 (0.97,1.05) | 0.8 |
| 4 years | 1256 | 1.02 (0.98,1.06) | 0.3 | 1.02 (0.99,1.06) | 0.5 | 1.02 (0.99,1.05) | 0.3 |
| 6 years | 1176 | 1.02 (0.99,1.06) | 0.2 | 1.01 (0.98,1.05) | 0.5 | 1.01 (0.98,1.04) | 0.4 |
| 7 years | 1217 | 1.02 (0.99,1.06) | 0.2 | 1.01 (0.97,1.04) | 0.7 | 1.02 (0.99,1.05) | 0.2 |
| 11 years | 1219 | 1.02 (0.99,1.05) | 0.2 | 0.99 (0.96,1.01) | 0.3 | 1.00 (0.98,1.03) | 0.8 |
| 15 years | 1134 | 1.01 (0.99,1.04) | 0.4 | 0.99 (0.96,1.01) | 0.2 | 1.01 (0.99,1.03) | 0.6 |
| 20 years | 1185 | 1.00 (0.97,1.03) | 0.9 | 0.99 (0.97,1.02) | 0.6 | 1.00 (0.97,1.02) | 0.7 |

Supplementary Table 7: Odds ratios (OR) for geometric remodelling categories compared with normal left ventricular structure for a 1 standard deviation conditional change in BMI and height: models adjusted for sex

|  |  | Eccentric hypertrophy | | Concentric hypertrophy | | Concentric remodelling | |
| --- | --- | --- | --- | --- | --- | --- | --- |
|  | n | OR (95% CI) | p-value | OR (95% CI) | p-value | OR (95% CI) | p-value |
| Change 2-4y  BMI (per SD)  Height (per SD) | 967 | 1.19 (0.97,1.47)  1.13 (0.91,1.40) | 0.1  0.3 | 1.07 (0.88,1.31)  1.03 (0.84,1.26) | 0.5  0.8 | 1.08 (0.91,1.27)  1.06 (0.91,1.26) | 0.4  0.5 |
| Change 4-7  BMI (per SD)  Height (per SD) | 1081 | 1.07 (0.88,1.29)  1.05 (0.86,1.29) | 0.5  0.6 | 1.13 (0.94,1.37)  0.96 (0.78,1.17) | 0.2  0.7 | 0.93 (0.79,1.09)  0.98 (0.83,1.15) | 0.4  0.8 |
| Change 7-15y  BMI (per SD)  Height (per SD) | 944 | 1.19 (0.96,1.46)  1.00 (0.82,1.21) | 0.1  0.96 | 1.35 (1.11,1.65)  0.91 (0.75,1.11) | 0.003  0.4 | 1.18 (0.99,1.40)  0.94 (0.80,1.10) | 0.06  0.4 |
| Change 15-20y  BMI (per SD)  Height (per SD) | 919 | 1.03 (0.84,1.26)  0.90 (0.74,1.11) | 0.8  0.3 | 1.17 (0.96,1.41)  0.89 (0.73,1.09) | 0.1  0.3 | 0.90 (0.76,1.06)  0.89 (0.76,1.06) | 0.2  0.2 |
