# Supplementary material for: Birthweight, childhood growth and left ventricular structure at age 60–64 years in a British birth cohort study
Source: Int J Epidemiol. 2016 Jul 13;45(4):1091–102. doi: 10.1093/ije/dyw150 (PMC5841632; doi:10.1093/ije/dyw150)
Supplement: Supplementary Data [file supp_45_4_1091__index.html]

Birthweight, childhood growth and left ventricular structure at age 60–64 years in a British birth cohort study — Supplementary Data 

# Birthweight, childhood growth and left ventricular structure at age 60–64 years in a British birth cohort study

## Supplementary Data

files

- Supplementary Data - docx file
